# K-Hammer percutaneous fixation: a novel technique for preventing iatrogenic ulnar nerve injury in pediatric supracondylar humeral fractures

**DOI:** 10.3389/fped.2025.1718195

**Published:** 2025-12-17

**Authors:** Yijun Zhou, Xiaoan Bai, Changhong Li, Mi Zhou, Fan Bai, Jiang Chen, Guanwen Sun

**Affiliations:** 1 The First People’s Hospital of Changde, Changde Hospital Affiliated to Xiangya Medical College of Central South University, Changde, Hunan, China; 2 Department of Orthopedics, Second Xiangya Hospital of Central South University, Changsha, China

**Keywords:** K-Hammer, iatrogenic ulnar nerve injury, pediatric supracondylar fracture, cross-pinning, Gartland classification

## Abstract

**Background:**

This study evaluated the therapeutic efficacy of a novel percutaneous Kirschner wire (K-wire) fixation combined with the K-Hammer technique in pediatric patients with supracondylar humeral fractures.

**Methods:**

This retrospective cohort study included 34 pediatric patients [13 males (38.24%); 21 females (61.76%); mean age 5.82 ± 2.54 years] with acute extension-type supracondylar humeral fractures (diagnosed ≤7 days post-trauma). Under general anesthesia, fractures underwent fluoroscopy-guided closed reduction and percutaneous fixation: two lateral-entry 1.5–2.0 mm K-wire provided initial stabilization, followed by a third medial-entry K-wire inserted using the K-Hammer technique to achieve a biomechanically optimized cross-pinning configuration. Postoperatively, the elbow was immobilized in a 90° functional position with a long-arm fiberglass cast for 4 weeks.

**Results:**

Over a mean follow-up of 12.24 ± 4.45 months (range: 6–23 months), functional outcomes per Flynn's criteria were excellent in 32 patients (94.12%), good in 2 (5.88%), and fair in 0 (0%). No cases of secondary displacement, osteonecrosis, or major complications—such as nonunion, iatrogenic neurovascular injury, myositis ossificans, or chronic elbow dysfunction—were observed during postoperative monitoring.

**Conclusions:**

The K-Hammer-assisted medial K-wire insertion provides a streamlined, reproducible approach for managing irreducible extension-type pediatric supracondylar humeral fractures. It effectively mitigates iatrogenic ulnar nerve injury, minimizes soft tissue trauma, ensures biomechanical stability, and promotes optimal long-term elbow kinematics.

## 1 Introduction

Supracondylar fractures of the humerus are the most common type of elbow fracture in children, accounting for 15%–20% of all pediatric fractures, with the highest incidence occurring in children aged 5–8 years (1). This type of fracture not only significantly impacts a child's daily life but also may lead to serious complications, such as neurovascular injuries and elbow dysfunction. In recent years, with changes in children's activity patterns and increased physical activity intensity, the incidence of supracondylar fractures has shown an increasing trend (2). Closed reduction and cross-K-wire fixation have become the gold standard for treating this type of fracture because of their simplicity, minimal invasiveness, and high stability in fracture reduction. However, with the widespread use of this technique, the incidence of iatrogenic ulnar nerve injury has gradually drawn the attention of clinicians.

This study aims to retrospectively evaluate the clinical application, technique, advantage, and limitations of K-Hammer percutaneous fixation in preventing iatrogenic ulnar nerve injury in extension-type pediatric supracondylar humeral fractures.

## 2 Materials and methods

### 2.1 Clinical data

In this retrospective, single-center study, we screened all pediatric patients who underwent surgical treatment for extension-type supracondylar humeral fractures at the Department of Orthopedics of The First People's Hospital of Changde between September 2020 and September 2024. Thirty-four patients (13 male, 21 female) aged 2–10 years met the eligibility criteria and were ultimately included. All injuries were sustained after low-energy falls and were classified as Gartland type III or IV patterns. No *a priori* sample size calculation was performed due to the retrospective, single-arm design, which is consistent with reporting standards for descriptive case series (3).

#### 2.1.1 Inclusion criteria

(1) Age 2–10 years (inclusive); (2) displaced, extension-type supracondylar humeral fracture (Gartland III–IV); (3) no preoperative clinical evidence of ulnar neuropathy; (4) intraoperative ultrasound confirming absence of ulnar nerve entrapment; (5) intact contralateral elbow for comparison. Extension-type was chosen due to its association with higher ulnar nerve injury risk (4); this narrow focus limits generalizability to extension-type fractures.

#### 2.1.2 Exclusion criteria

(1) Open fractures; (2) preoperative physical examination demonstrating ulnar nerve deficit or intraoperative ultrasound revealing ulnar nerve entrapment; (3) concomitant fractures of other anatomic regions; (4) pathological or multiple fractures.

### 2.2 Surgical technique

After successful induction of general anesthesia, the patient was placed in the supine position with the affected limb positioned at the edge of the operating table to allow full C-arm fluoroscopic visualization. Routine sterile draping was then applied.

An assistant applied gentle manual longitudinal traction to the forearm for 30–60 s, while the surgeon simultaneously provided countertraction at the ipsilateral shoulder, effectively correcting angular deformities in both the coronal and sagittal planes.

Closed reduction was then performed under fluoroscopic guidance using a combination of forearm supination, varus or valgus correction (depending on the fracture displacement pattern), and gentle axial compression, with the elbow maintained in flexion. The quality of reduction was confirmed by the restoration of the teardrop sign, Baumann angle, and humerocapitellar angle on anteroposterior and lateral fluoroscopic views.

Once satisfactory reduction was achieved, two 1.5–2.0 mm Kirschner wires (K-wires) were inserted percutaneously from the lateral humeral condyle in a divergent configuration to provide initial stabilization. Fluoroscopy confirmed appropriate K-wire placement and fracture stability.

**Figure 1:**
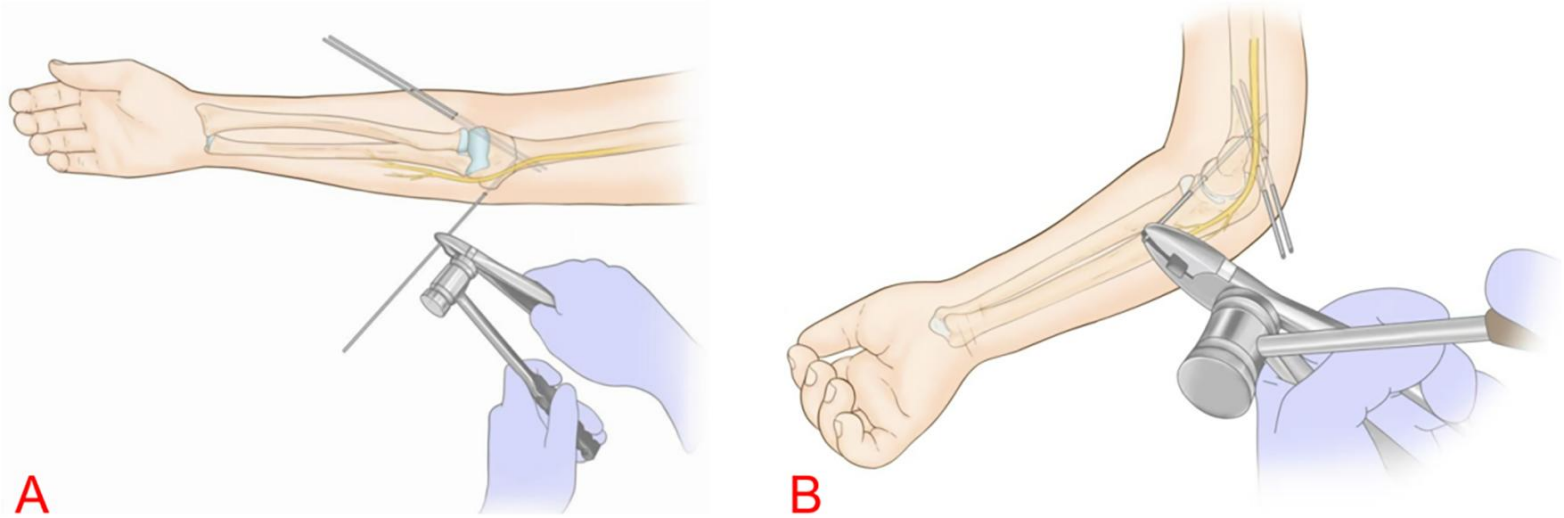
This figure present hand-drawn illustrations of the K-hammer surgical technique. **(A)** Shows the hand-drawn illustration of Kirscher wire insertion at the medical condyle of the humerus in the anteroposterior view of the elbow joint; **(B)** shows the hand-drawn illustration of Kirschner wire insertion at the medical condyle of the humerus in the lateral view of the elbow joint.

**Figure 2:**
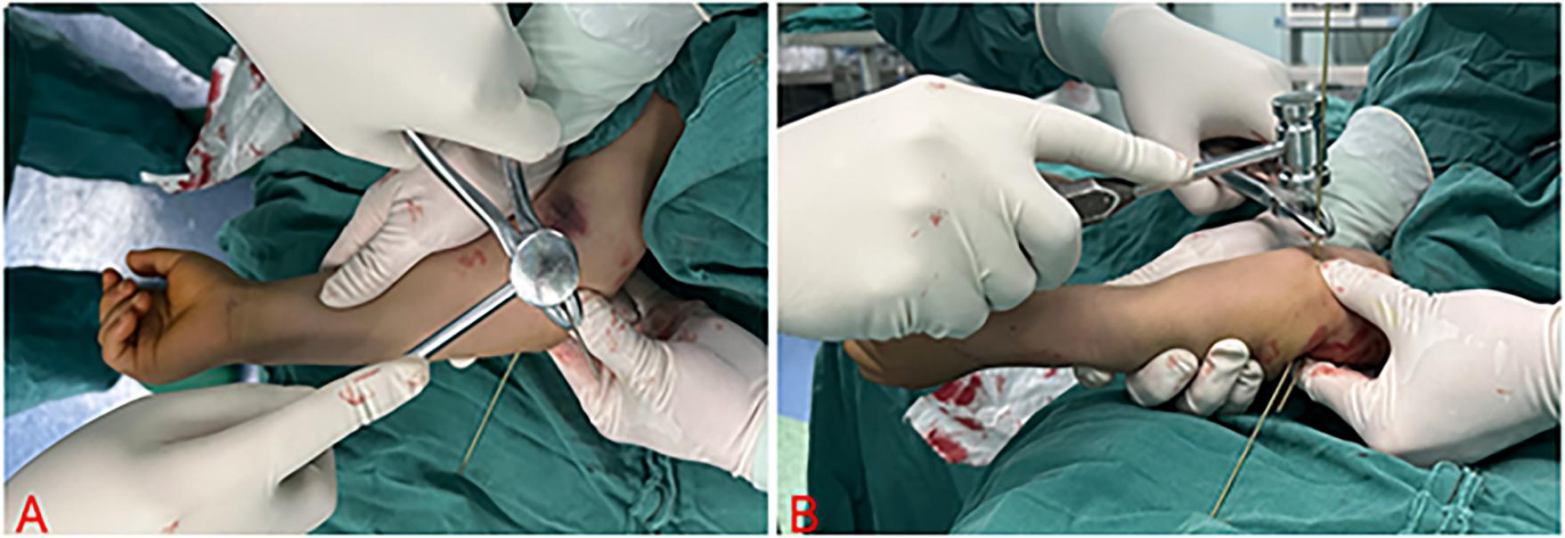
This figure present intraoperative photographs of the K-hammer surgical technique. **(A)** Shows photograph of Kirscher wire insertion at the medical condyle of the humerus in the anteroposterior view of the elbow joint; **(B)** shows photograph of Kirschner wire insertion at the medical condyle of the humerus in the lateral view of the elbow joint.

**Figure 3:**
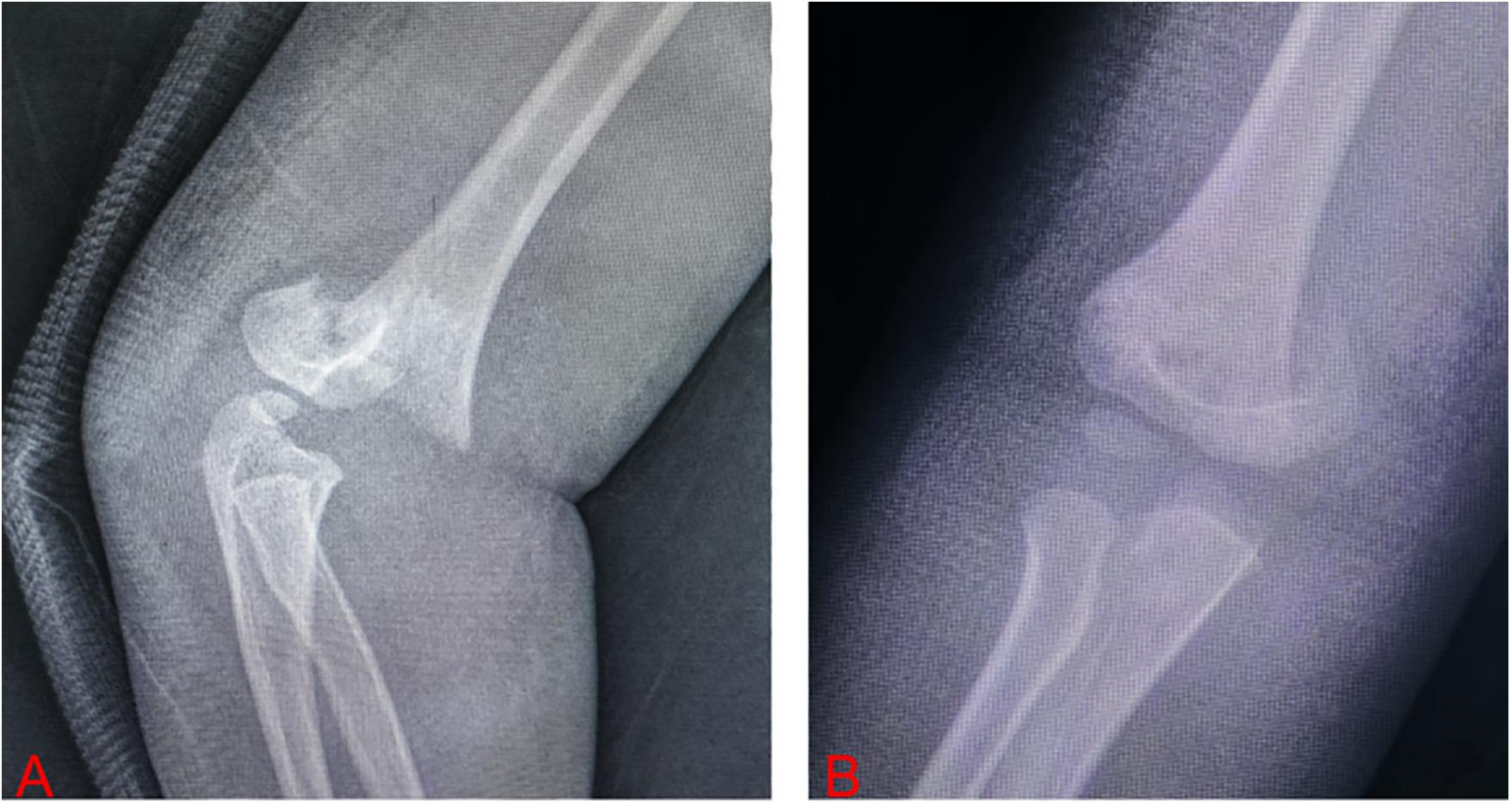
A 4-year-old female patient with a Gartland type III supracondylar humeral fracture. **(A)** Shows lateral x-ray of the elbow joint, and **(B)** shows the anteroposterior x-ray of the elbow joint.

**Figure 4:**
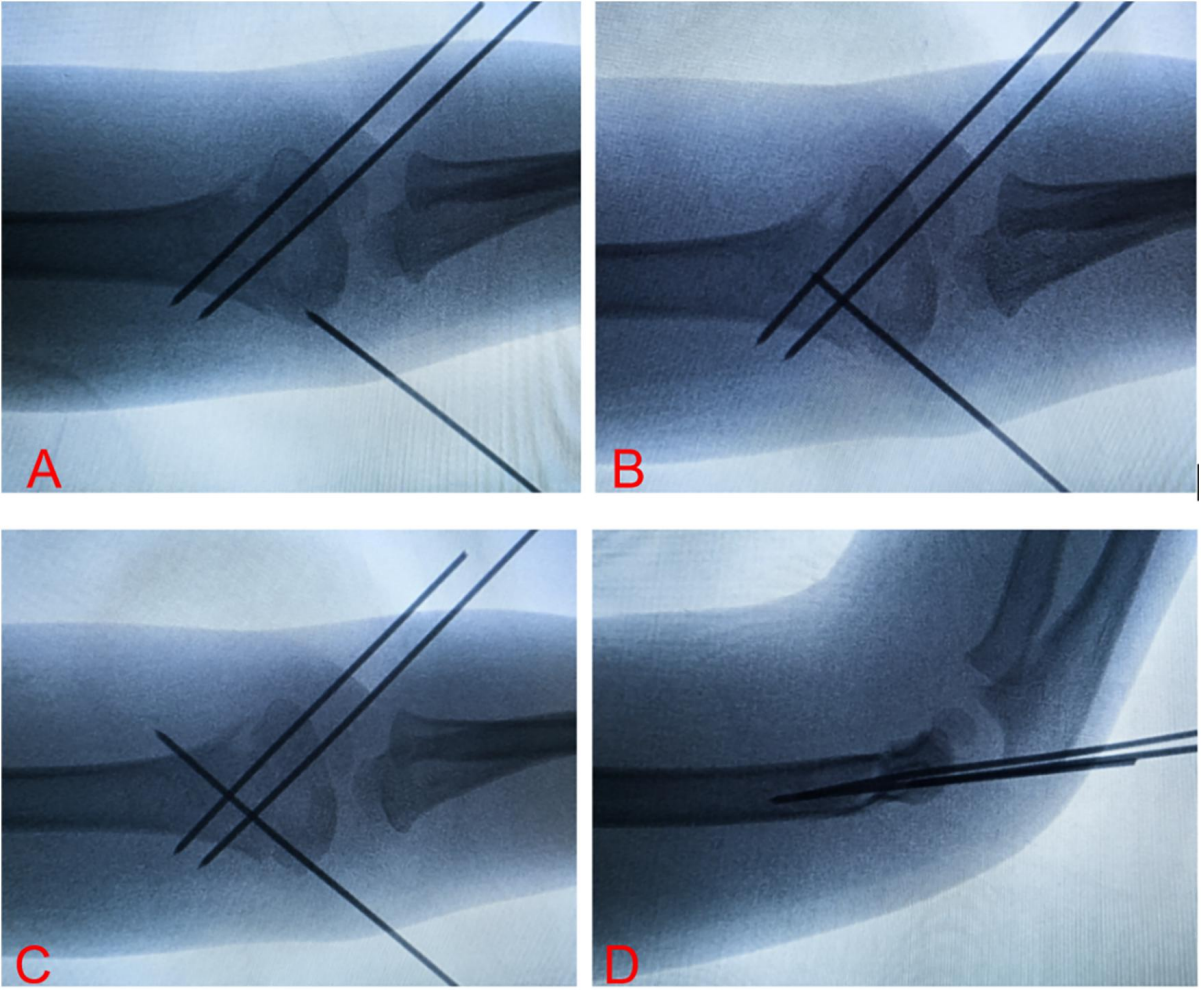
This figure shows the K-hammer surgical procedure under the guidance of a C-arm. **(A,B)** Show the process of slow Kirscher wire insertion at the medical condyle of the humerus, and **(C,D)** show the anteroposterior and lateral x-rays of the elbow joint after the completion of Kirschner wire insertion.

The surgeon then maintained the elbow in slight extension (approximately 30°–60°) to promote posterior displacement of the ulnar nerve. Using the left thumb, the surgeon applied firm posterior pressure on the medial epicondyle to further displace the ulnar nerve posteriorly. The K-wire entry point was selected slightly anterior to the medial epicondyle. The wire was advanced perpendicularly through the medial cortex using a bone hammer securely held with bone-holding forceps to prevent rotation, thereby minimizing the risk of nerve entanglement. Upon contact with the opposite cortex, a low-speed electric drill was used to complete transfixation and avoid thermal injury. Final K-wire positions and fracture alignment were verified fluoroscopically (Figures 1–4).

All K-wires were bent and trimmed, leaving approximately 1.5 cm protruding through the skin for ease of postoperative removal. Pin sites were disinfected with 10% povidone-iodine and covered with sterile dressings. The elbow was immobilized in a long-arm fiberglass cast at 90° of flexion with the forearm in neutral rotation for four weeks.

## 3 Results

### 3.1 Demographic and baseline surgical characteristics

A total of 34 pediatric patients (mean age 5.82 ± 2.54 years) with acute extension-type supracondylar humeral fractures were included in this study. The cohort comprised 13 males (38.24%) and 21 females (61.76%), with a mean hospital stay of 4.12 ± 1.68 days. The mean operative time was 44.53 ± 7.44 min, and the mean follow-up period was 12.24 ± 4.45 months (range: 6–23 months) (Table 1).

**Table 1:** Baseline demographic and clinical characteristics of the study cohort (*n* = 34).

Variable	Value
Continuous variables	Mean ± SD
Age (years)	5.82 ± 2.54
Hospital stay (days)	4.12 ± 1.68
Operative time (minutes)	44.53 ± 7.44
Follow-up duration (months)	12.24 ± 4.45
Radiographic parameters	
Baumann angle (°)	78.38 ± 4.96
Humerocapitellar angle (°)	33.75 ± 8.79
Functional outcomes	
Flexion (°)	144.26 ± 9.02
Extension deficit (°)	0.85 ± 2.24
Carrying angle, affected limb (°)	12.29 ± 5.08
Carrying angle, unaffected limb (°)	13.91 ± 2.19
Categorical variables	*n* (%)
Sex	
Male	13 (38.24%)
Female	21 (61.76%)
Injured side	
Left	21 (61.76%)
Right	13 (38.24%)
Gartland classification	
Type III	23 (67.65%)
Type IV	11 (32.35%)
Time to radiographic union (weeks)	5.2 ± 1.1 weeks (range: 4–8 weeks)

All radiographic angles were measured by a single blinded observer using digital calipers on PACS; intra-observer reliability (ICC) was 0.92 for Baumann angle and 0.89 for humerocapitellar angle, consistent with methodology recommended for pediatric elbow fracture assessment (5).

### 3.2 Radiographic parameters

Preoperative and postoperative radiographic assessments (Baumann and humerocapitellar angles) were measured by a single blinded observer using digital tools, with duplicate measurements 2 weeks apart for intra-observer reliability, which demonstrated clear correction of key anatomical parameters toward normal reference ranges (Baumann angle: 70°–80°; humerocapitellar angle: 30°–40°) (5). The mean Baumann angle was corrected to 78.38 ± 4.96°, whereas the humerocapitellar angle improved to 33.75 ± 8.79°. The postoperative elbow range of motion (ROM) showed excellent restoration, with mean flexion achieving 144.26 ± 9.02° (deficit: 1.5 ± 2.0° vs. contralateral side) and extension limited by only 0.85 ± 2.24°. The carrying angle of the affected limbs (12.29 ± 5.08°) deviated minimally from that of the unaffected limbs (13.91 ± 2.19°). Functional Outcomes According to Flynn's criteria, functional assessment revealed excellent outcomes in 32 patients (94.12%) with flexion–extension limitations within 0°–5°. Two patients (5.88%) demonstrated good outcomes with a 6°–10° limit, whereas no patients presented fair or poor results. Comparative analysis of the carrying angles between the affected and unaffected limbs revealed that 30 patients (88.24%) maintains optimal alignment with 0°–5° loss, and 4 patients (11.76%) exhibited 6°–10° loss. No patients experienced loss greater than 10° (Table 2).

**Table 2:** Functional outcomes according to Flynn's criteria.

Outcome	Excellent	Good	Fair	Poor
Elbow joint function	Flexion and extension are limited by 0°–5°	Flexion and extension are limited by 6°–10°	Flexion and extension are limited by 11°–15°	Flexion and extension are limited by more than 15°
Number of cases	32	2	0	0
Comparison of carrying angles between affected and unaffected limbs	Loss of 0°–5°	Loss of 6°–10°	Loss of 11°–15°	Loss of greater than 15°
Number of cases	30	4	0	0

### 3.3 Complication profile

No cases of secondary displacement, osteonecrosis, nonunion, iatrogenic neurovascular injury, myositis ossificans, or chronic elbow dysfunction were observed during the follow-up period.

## 4 Discussion

Current biomechanical evidence indicates that primary stabilization of the medial column during crossed-pin fixation for supracondylar humeral fractures confers enhanced construct rigidity, particularly in comminuted or highly unstable fracture configurations (6). Nonetheless, the persistent risk of iatrogenic ulnar nerve complications—including traction injuries and thermal damage during trans-epicondylar pin placement—remains a critical limiting factor for its universal implementation. Current evidence from our clinical study demonstrates that the lateral-entry-first technique offers superior biomechanical and clinical benefits. The narrow inclusion of only extension-type fractures limits applicability to the more common extension-type, which may have different biomechanical demands.

First, the lateral-entry-first approach provides optimal fracture stabilization while minimizing iatrogenic risk. As shown in our series, the initial placement of two lateral-entry K-wires established primary stability without jeopardizing neurovascular structures. This technique allows for better control of reduction quality under fluoroscopic guidance before engaging the medial column. Notably, the risk of ulnar nerve injury is significantly reduced when the medial pin is placed last, as the nerve is naturally displaced posteriorly during elbow extension.

Second, contrary to previous reports, our data indicate that the lateral-entry-first technique does not prolong the surgical time, with a mean operative duration of 44.53 ± 7.44 min. The initial lateral fixation actually streamlines the subsequent medial pin placement by providing a stable reference point, eliminating the need for repeated adjustments typically required in medial-first approaches. This technical modification maintains soft tissue integrity while ensuring optimal biomechanical stability.

Furthermore, the biomechanical advantages of lateral-entry-first fixation are evident in our radiographic outcomes. The construct comprising initial lateral pins followed by a carefully placed medial pin demonstrated excellent maintenance of reduction, with the Baumann angle restored to 78.38 ± 4.96° and the humerocapitellar angle improved to 33.75 ± 8.79°. This optimized cross-pinning configuration effectively prevented secondary displacement without compromising adjacent anatomical structures.

In conclusion, while medial pinning first has been traditionally favored, the lateral-entry-first technique offers distinct advantages in terms of safety, efficiency, and biomechanical stability. This approach particularly excels in minimizing iatrogenic complications while maintaining optimal fracture reduction and elbow kinematics (7–9).

The positioning of the elbow during medial pin placement plays a critical role in minimizing the risk of ulnar nerve injury. Shih CA et al. (10) demonstrated a significant correlation between the elbow flexion angle and ulnar nerve subluxation, with anterior subluxation occurring in 53.3% of patients at 120° flexion, 40% at 90° flexion, and 16.7% at 60° flexion, with no subluxation observed at 30° flexion. This finding is particularly relevant given the intimate anatomical relationships among the ulnar nerve, cubital tunnel, and medial epicondyle (11, 12).

Several studies have documented the prevalence of ulnar nerve instability in pediatric populations. Zaltz et al. (13) reported that 54.8% (28/52) of children aged 6–10 years presented ulnar nerve instability. Furthermore, ultrasound examination of 237 children revealed that elbow flexion to 90° or 120° resulted in ulnar nerve subluxation or dislocation from the cubital tunnel in 40%–58% of cases. These findings highlight the potential risks associated with excessive elbow flexion during medial pin placement.

While excessive elbow flexion is commonly employed to maintain reduction in extension-type supracondylar humerus fractures, this position significantly increases the risk of ulnar nerve injury during medial pin insertion. Intraoperative ultrasound monitoring conducted during fracture reduction and fixation (14) revealed that among 15 children with excessive elbow flexion, all demonstrated anterior ulnar nerve subluxation. Successful medial pin placement required elbow extension to approximately 90°.

On the basis of these findings, we recommend maintaining the elbow at approximately 30° of extension when performing medial K-wire) fixation for supracondylar humerus fractures, particularly in cases of ulnar nerve dislocation. This positioning effectively reduces the risk of iatrogenic ulnar nerve injury by preventing nerve dislocation during the procedure. The technical modification using K-Hammer-assisted medial K-wire insertion further enhances safety by maintaining soft tissue integrity while ensuring adequate biomechanical stability at the fracture site.

The implementation of the biomechanically optimized cross-pinning construct for extension-type fractures, comprising two lateral-entry K-wires and one medial-entry wire, provided adequate stability at the fracture site. Findings are specific to extension-type but may be generalizable to extension-type pending further research. This configuration facilitated optimal long-term restoration of elbow joint kinematics without compromising adjacent anatomical structures. Radiographic union was achieved at a mean of 5.2 ± 1.1 weeks (range: 4–8 weeks), consistent with typical healing timelines for pediatric supracondylar humeral fractures (Table 2).

Our study demonstrated that the biomechanically optimized cross-pinning construct, consisting of two lateral-entry K-wires and one medial-entry wire deployed through the K-Hammer technique, provides superior fracture stability while maintaining an excellent safety profile. This finding aligns with previous biomechanical research showing that crossed fixation offers significantly better stability than lateral fixation alone under various loading conditions (15). The technical modification using K-Hammer-assisted medial K-wire insertion effectively eliminates iatrogenic ulnar nerve injury while maintaining soft tissue integrity, which represents a significant advancement in surgical technique.

The implementation of our modified cross-fixation technique yielded excellent functional outcomes in 94.12% of the patients, with only 5.88% demonstrating good outcomes and no patients showing fair or poor results according to Flynn's criteria. These results contrast with those of Brauer et al.'s systematic review (16), which reported a 40% lower rate of loss of reduction with crossed fixation than with lateral fixation. In comparison to 3 lateral pinning techniques (16), our K-Hammer method provides enhanced stability similar to crossed fixation but with reduced ulnar nerve risk. Mini-approach medial pinning (17) offers direct visualization but increases soft tissue trauma, unlike our percutaneous approach. Our series demonstrated no instances of secondary displacement, further confirming the superior stability of this construct.

Regarding functional recovery, our results show that the crossed fixation group achieved excellent elbow motion restoration (mean flexion 144.26 ± 9.02°) and minimal carrying angle deviation (mean 12.29 ± 5.08°), which is consistent with the findings of Na et al.'s meta-analysis (18). The technical modification of the K-Hammer technique with careful soft tissue protection effectively reduced the risk of iatrogenic ulnar nerve injury, as Woratanarat et al. (17) suggested through mini-open incision techniques.

The long-term follow-up results from our study confirm the advantages of cross-fixation in preventing abnormal fracture healing. The maintenance of the Baumann angle (78.38 ± 4.96°) and humerocapitellar angle (33.75 ± 8.79°) demonstrates the effectiveness of this technique in preventing deformities such as cubitus varus, supporting Kawak et al.'s findings (19). Radiographic union was achieved at a mean of 5.2 ± 1.1 weeks (range: 4–8 weeks), consistent with typical healing timelines for pediatric supracondylar humeral fractures.

Importantly, our cohort demonstrated a complication-free safety profile over a mean follow-up of 12.24 months. No instances of iatrogenic ulnar nerve injury or other major neurovascular complications were recorded—a notable achievement given that ulnar nerve injury is reported in 1%–11% of conventional cross-pinning procedures (4). In contrast to historical series employing three lateral pins or mini-open medial approaches (8), our percutaneous K-Hammer-assisted technique achieved comparable or superior safety outcomes without requiring additional surgical incisions. This suggests that the K-Hammer modification not only preserves soft tissue integrity but also effectively mitigates the risk of iatrogenic nerve injury, even in high-risk extension-type fractures.

Our study has several limitations that warrant acknowledgment. First, it is a single-center, retrospective cohort study without a control group (e.g., patients treated with 3 lateral pinning or mini-open medial pinning), which precludes direct comparative analysis of complication rates or functional outcomes. Second, the relatively small sample size (*n* = 34) limits statistical power for subgroup analyses and may affect the generalizability of our findings. Third, although all surgeries were performed by experienced surgeons, the learning curve associated with the K-Hammer technique was not formally assessed. Future prospective, multicenter studies with larger cohorts and control groups are needed to validate our results and further evaluate the safety and efficacy of this novel technique. Notably, the technique assumes surgeon familiarity with fluoroscopic guidance; novice surgeons should practice on models before clinical application.

## 5 Conclusions

In conclusion, K-Hammer-assisted percutaneous medial pinning provides a safe, reproducible, and biomechanically stable method to prevent iatrogenic ulnar nerve injury in pediatric extension-type supracondylar humeral fractures, with excellent functional outcomes and minimal soft tissue disruption.

## Data Availability

The raw data supporting the conclusions of this article will be made available by the authors, without undue reservation.
